# Bonding Effectiveness of Veneering Ceramic to Zirconia after Different Grit-Blasting Treatments

**DOI:** 10.3390/dj12070219

**Published:** 2024-07-15

**Authors:** Francesca Zicari, Carlo Monaco, Marcio Vivan Cardoso, Davide Silvestri, Bart Van Meerbeek

**Affiliations:** 1 KU Leuven, Department of Oral Health Sciences, Biomaterials—BIOMAT & UZ Leuven, 3000 Leuven, Belgium; 2 University of Modena and Reggio Emilia, Department of Surgery, Medical, Dentistry and Morphological Sciences with Transplant Interest, Oncology and Regenerative Medicine (CHIMOMO), 41121 Modena, Italy

**Keywords:** zirconia, veneering ceramic, surface treatments, grit-blasting, surface roughness, micro-tensile bond strength

## Abstract

**Objective:** To determine the effect of grit-blasting before and after sintering on the surface roughness of zirconia and the micro-tensile bond strength of a pressable veneering ceramic to zirconia. **Methods:** Pre-sintered zirconia blocks (IPS e.max ZirCAD, Ivoclar) were divided into four test groups of three specimens each and a control group (‘CTR’; no surface treatment). Pre-S-30, Pre-S-50, and Pre-S-110 were grit-blasted with 30-µm SiO_2_-coated Al_2_O_3_, 50-µm Al_2_O_3_ and 110-µm Al_2_O_3_ particles, respectively, before sintering. Post-S-30 was grit-blasted with 30-µm SiO_2_-coated Al_2_O_3_ after sintering. For each treatment, the surface roughness was measured (Ra, Perthometer M4P, Mahr Perthen). After sintering the zirconia blocks, a liner was applied and a pressable ceramic (IPS e.max ZirPress, Ivoclar) was heat-pressed. Sixteen microbars were obtained from each block and submitted to micro-tensile bond-strength (µTBS) testing. Data were analyzed with one-way ANOVA. Any correlation between Ra and µTBS was evaluated (Sperman test). **Results:** Grit-blasting before sintering with 110-µm Al_2_O_3_ (Ra_Pre-S-110_ = 3.4 ± 0.4 µm), 50-µm Al_2_O_3_ (Ra_Pre-S-50_ = 2.3 ± 0.5 µm), and 30-µm SiO_2_-coated Al_2_O_3_ (Ra_Pre-S-30_ = 1.2 ± 0.2 µm) resulted in significantly higher roughness than grit-blasting after sintering with 30-µm SiO_2_-coated Al_2_O_3_ (Ra_Post-S-30_ = 0.5 ± 0.1 µm). The highest µTBS was measured when the sintered zirconia was grit-blasted with 30-μm SiO_2_-coated Al_2_O_3_ (µTBS_Post-S-30_ = 28.5 ± 12.6 MPa), which was significantly different from that of specimens that were grit-blasted before sintering (µTBS_Pre-S-30_ = 21.8 ± 10.4; µTBS_Pre-S-50_ = 24.1 ± 12.6; µTBS_Pre-S-110_ = 26.4 ± 14.1) or were not grit-blasted (µTBS_CTR_ = 20.2 ± 11.2). **Conclusions:** Grit-blasting zirconia before sintering enhanced the surface roughness proportionally to the particle size of the sand used. Grit-blasting with 30-µm SiO_2_-coated Al_2_O_3_ after sintering improved bonding of the veneering ceramic to zirconia. **Clinical Significance**: As grit-blasting with 30-µm SiO_2_-coated Al_2_O_3_ after sintering improved bonding of the veneering ceramic to zirconia, it may reduce veneering ceramic fractures/chipping.

## 1. Introduction

In the last few decades, the use of zirconia in prosthodontics has been widened thanks to its superb aesthetics, excellent mechanical and optical properties, and high biocompatibility [1,2,3]. Nowadays all-ceramic zirconia restorations are commonly used in the anterior and posterior region as an effective alternative to porcelain-fused-to-metal restorations (PFMs) [3,4]. The higher mechanical performance of yttria-stabilized tetragonal zirconia polycrystal (Y-TZP) zirconia combined with CAD/CAM fabrication and digital workflows allow single crowns, fixed partial dentures (FPDs), and implant-supported restorations to be realized with high accuracy and success rate [5,6,7,8,9].

To achieve better aesthetic results, zirconia frameworks can be veneered with porcelain, which is accurately layered to provide the final restoration exclusive aesthetic characteristics that can barely be distinguished from the neighboring natural teeth [5,10,11]. Alternatively, ceramic can be pressed onto zirconia frameworks as individual patient-specific characterization. Although heat pressing is more laborious, it involves a one-step layering procedure and avoids interfacial porosities thanks to the lost-wax technique [12,13].

However, establishing a strong and durable bond of veneering ceramic to 3Y-TZP appeared challenging [14,15,16], because delamination and chipping may occur during function [17,18,19]. Overall, although a high survival and success rate of all-ceramic restorations have been reported at 5 to 10 years, clinical studies reported a failure rate in the range of 10–15% after five years for veneered Y-TZP frameworks, this due to chipping of the ceramic veneer [7,14,15,17,18].

As explained by Aboushelib et al. [5,10], a crack initiated at the ceramic-zirconia interface can grow through the weakest layer because of asymmetric stress distribution in the specimen. Therefore, traces of elements may be left attached to the interface. When analyzed, this can erroneously be interpreted as cohesive failure. Moreover, the initial point of stress concentration and crack growth is often difficult to determine.

Different treatments and techniques have been proposed to improve bonding at the veneering ceramic-zirconia interface, including air-abrasion with aluminum oxide (Al_2_O_3_), silica tribochemical coating, liner application, acid etching, or plasma treatment [20,21,22,23,24,25,26]. Silica tribochemical coating has been proven to improve the bonding of luting agents to zirconia, particularly when the CoJet system (3M Oral Care, Seefeld, Germany) was used [19]. This system uses silica-coated alumina particles for grit-blasting, hereby depositing silica onto the surface by means of high spot-heating produced by the blasting pressure, by which it enables additional silanization. Since silicate-based veneering porcelains are often used to bond to zirconia frameworks, silica-coating zirconia might enhance the bond strength of the veneering ceramic to zirconia as well. However, whether silica-coating could be effective to improve bonding at the veneering ceramic-zirconia interface has not yet been evaluated extensively.

In general, shear or micro-tensile bond-strength tests are used to measure bonding effectiveness. However, a shear bond-strength test may lead to non-interfacial stress distribution, inducing cohesive failures and erroneous interpretation of data. In particular, the micro-tensile bond-strength test (µTBS) has been proven to be a reliable test to evaluate bond strength of resin-based materials to a variety of substrates [27,28].

The aim of this study was to determine the effect of grit-blasting before and after sintering on surface roughness of zirconia and µTBS of veneering ceramic to zirconia.

The null hypotheses tested were that grit-blasting with Al_2_O_3_ or silica-coated Al_2_O_3_ particles before or after sintering does not affect (1) the surface roughness of zirconia and (2) the µTBS of the veneering ceramic fired onto zirconia.

## 2. Materials and Methods

### 2.1. Specimen Preparation

**Figure 1:**
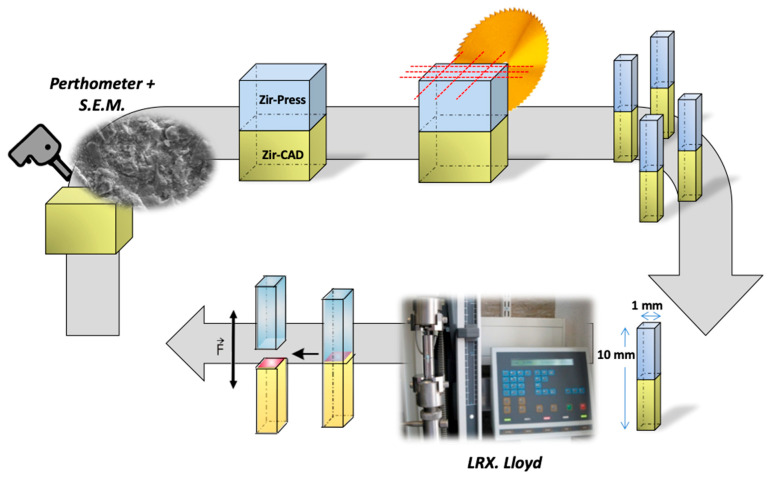
Specimen preparation.

Three zirconia blocks, namely ZirCAD C15 L (Ivoclar, Schaan, Liechtenstein), were sectioned into three smaller blocks using a low-speed diamond disc (MDS100, Norton, VA, USA). In total, 15 small blocks of 7.2-mm height, 9.2-mm width, and 9.2-mm length were cut. The 15 blocks were further subdivided into 5 groups of three specimens each depending on the surface treatment at the veneering ceramic/3Y-TZP interface (Figure 1).

#### 2.1.1. Surface Treatment

Four more groups of three specimens each were grit-blasted before or after sintering by applying the following procedures: ‘Pre-S-30’, ‘Pre-S-50’, and ‘Pre-S-110’ were grit-blasted with 30-µm SiO_2_-coated Al_2_O_3_ (CoJet, 3M Oral Care), 50-µm Al_2_O_3_, and 110-µm Al_2_O_3_ (Cobra, Renfert, Hilzingen, Germany) particles, respectively, before sintering; ‘Post-S-30’ was grit-blasted with 30-µm SiO_2_-coated Al_2_O_3_ (CoJet, 3M Oral Care) particles after sintering. One group did not receive any treatment (‘CTR’: no surface treatment); these specimens were only polished.

All specimens were grit-blasted using the same pressure of 2 bar for 15 s, with a 1-cm distance between nozzle and surface for the 30-µm SiO_2_-coated Al_2_O_3_ (CoJet, 3M Oral Care) and a 1.5-cm distance for the 50-µm and 110-µm alumina.

The materials tested and their properties are summarized in Table 1. Surface treatments evaluated in this study are presented in Table 2.

#### 2.1.2. Surface-Roughness Evaluation

Surface roughness (Ra) was measured using contact profilometry (Perthometer M4P, Mahr Perthen, Providence, RI, USA) of the polished, sandblasted, and silica-coated surface of each specimen. The surface was scanned twice by five parallel tracings with 1.0-mm intervals, upon which Ra was recorded.

#### 2.1.3. Over-Pressing Technique

A layer of IPS e.max ZirLiner (Ivoclar) was applied on the zirconia blocks and fired at 960 °C (Sintramat, Ivoclar), following the manufacturer’s instructions. Then, the veneering ceramic IPS e.max ZirPress (Ivoclar) was heat-pressed on top. A wax-up was performed using a coping in order to fabricate an equivalent veneering structure for the corresponding ZirCAD specimen. The wax surface was smoothed, finished, and invested using a special investing material (IPS PressVEST, Ivoclar) in a size-2 muffle according to the manufacturer’s instructions.

The wax was burned out and the muffle was heated. Copings were pressed using porcelain with the proper coefficient of thermal expansion (IPS e.max ZirPress, Ivoclar). After cooling, the investment was removed using a grit-blasting unit (Eurosab, Tissi, San Donato Milan, Italy) using 50-µm glass beads at 2-bar pressure. The reaction layer formed during pressing was removed by soaking the crowns in HF solution (IPS e.max Press Invex Liquid, Ivoclar) in an ultrasonic cleaner (Sonorex, Bandelin, Berlin, Germany) for 5 min. Blocks were then cleaned with running water for 3 min and dried. Pressing sprues and extrusion flushes were removed using a water-cooled air-turbine without pressure to protect the porcelain from heat damage.

### 2.2. Micro-Tensile Bond-Strength Test

The 15 IPS e.max ZirCAD/ZirPress (Ivoclar) blocks were stored in distilled water at 37 °C for 1 week. Afterwards, they were cut using a diamond-coated blade (Accutom-50, Stuers, Ballerup, Denmark) for sintered zirconia under water cooling, this to obtain 16 microbars out of each ceramic block. Each microbar had a length of 10 mm (5-mm ZirCAD and 5-mm ZirPress, Ivoclar) and a horizontal cross section of 1 mm^2^. In total, 48 specimens were obtained for each group. Microbars were attached to the testing unit (LRX, Lloyd, Hampshire, UK) using glue (Model Repair II Blue, Dentsply-Sankin, Ohtawara, Japan), taking care to exactly center the veneering ceramic-zirconia interface on the free space of the attachment unit. Specimens were loaded to failure at a crosshead speed of 1 mm/min. The maximum load at failure (N and MPa) was extracted from computer-generated files.

### 2.3. Microstructural Analysis by Stereomicroscopy and Scanning Electron Microscopy

Before pressing the veneering ceramic, specimens belonging to all pre/post-sintered 3Y-TZP blocks and CTR were gold-sputtered and examined using a scanning electron microscope (SEM, Zeiss EVO 40, D) equipped with an energy-dispersive X-ray analyzer (EDS, Inca, Oxford Instruments, Oxford, UK).

An analysis of the fractured specimens was carried out immediately after the µTBS test using a stereomicroscope (Wild M5A, Heerbrugg, Switzerland) at 25× magnification. Failures were classified as cohesive (within the veneering ceramic), adhesive (at the interface between veneering ceramic and zirconia), and mixed. In addition, randomly selected failed microbars were cleaned in an ultrasonic bath, gold-sputtered, and analyzed using SEM and EDS.

### 2.4. Statistical Analysis

Statistical analysis was performed using the software package Statistica (StatSoft 9.1, Tulsa, OK, USA).

One-way ANOVA with Tukey-HSD for post-hoc comparison was used to analyze surface roughness (Ra, *p* < 0.05) and µTBS (*p* < 0.05). The µTBS data were statistically analysed either excluding specimens that failed prematurely during the µTBS test (referred to as pretesting failures, PTF), either including them as the lowest measured value or including them as 0 MPa.

The Spearman test was used to evaluate any correlation between µTBS and Ra (*p* < 0.05).

## 3. Results

**Figure 2:**
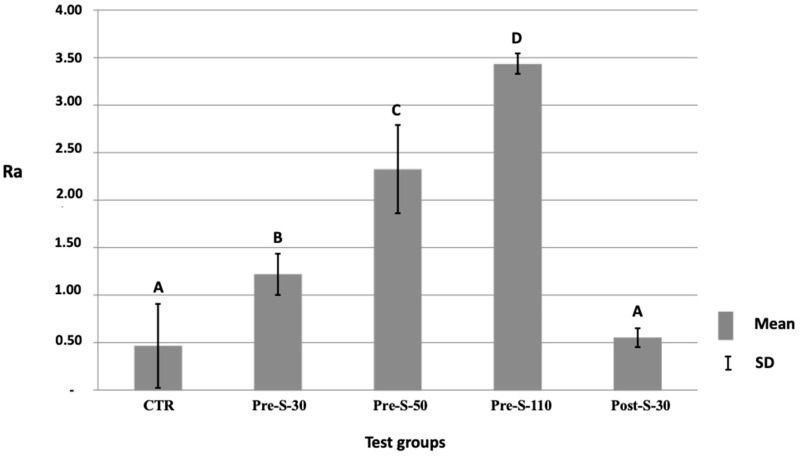
Graph showing means and standard deviation of surface roughness (Ra) for all surface treatments tested (One-way ANOVA with Tukey-HSD for post-hoc comparison). Different capital letters indicate statistically significant differences.

Regarding surface roughness (Ra), specimens grit-blasted with 110-µm (Ra_Pre-S-110_ = 3.4 ± 0.4 µm), 50-µm (Ra_Pre-S-50_ = 2.3 ± 0.5 µm), and 30-µm (Ra_Pre-S-30_ = 1.2 ± 0.2 µm) (SiO_2_-coated) Al_2_O_3_ particles were significantly rougher than the control specimens that were not grit-blasted (Ra_CTR_ = 0.5 ± 0.1 µm) and the specimens that were grit-blasted after sintering (Ra_Post-S-30_ = 0.5 ± 0.1 µm) (*p* < 0.05). Specimens grit-blasted with 110-µm Al_2_O_3_ were most rough. Ra for all groups is presented in Figure 2.

**Figure 3:**
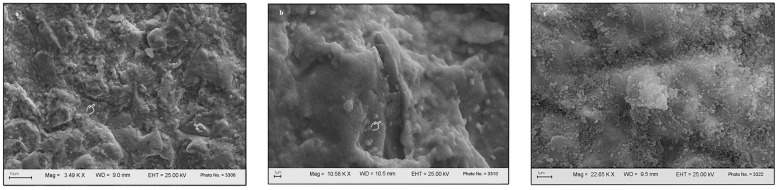
SEM photomicrographs of grit-blasted specimens. (**a**) Zirconia surface grit-blasted with 30-µm SiO_2_-coated Al_2_O_3_ before sintering. (**b**) Zirconia surface grit-blasted with 110-µm Al_2_O_3_ before sintering. The pointers indicate darker alumina particles fractured at grit-blasting impact and melted onto the zirconia surface. (**c**) Zirconia surface grit-blasted with 30-µm SiO_2_-coated Al_2_O_3_ after sintering. The tiny particles represent silica deposited on the surface.

The grit-blasted surfaces presented detachments and plastic deformation of the material (Figure 3a). Grit-blasting before sintering also induced chemical changes, as detected by EDS (Figure 3a,b), whereas specimens grit-blasted after sintering revealed several fine silica particles deposited by CoJet (3M Oral Care) onto the zirconia surface (Figure 3c).

The highest µTBS was recorded when zirconia blocks were grit-blasted with 30-µm SiO_2_-coated Al_2_O_3_ using CoJet (3M Oral Care) after sintering. When the pretesting failures were included as 0 MPa (‘PTF = 0’) or as the lowest measured value (‘PTF = MIN VALUE’), Post-S-30 performed significantly better than the control (CTR) (*p* < 0.05). Grit-blasting before sintering did not significantly improve bond strength. However, a trend of increasing bond strength proportional to the size of the airborne particles was observed.

**Figure 4:**
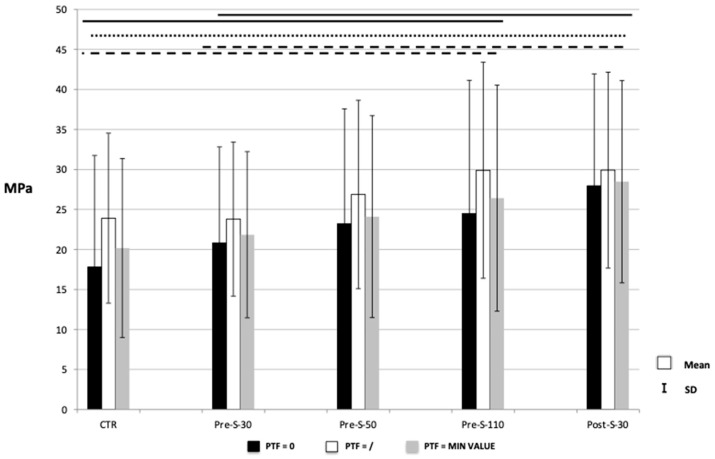
Graph showing the mean micro-tensile bond strength (µTBS) with standard deviation for all experimental groups. Connected lines indicate absence of significant difference. Different lines refer to the different strategies for dealing with pretesting failures (PTF). The continuous black lines refer to the analysis conducted by including PTF with the lowest measured value (‘PTF = MIN VALUE’). The dotted black line refers to the analysis conducted by excluding PTF (‘PTF = /’). The dashed black line refers to the analysis conducted by including PTF as 0 MPa (‘PTF = 0’).

The mean µTBS with standard deviation and failure patterns are presented in Figure 4 and Table 3.

The number of pretesting failures observed for each group, out of a total number of 144 specimens (48 specimens within each group), is also presented in Table 3.

**Figure 5:**
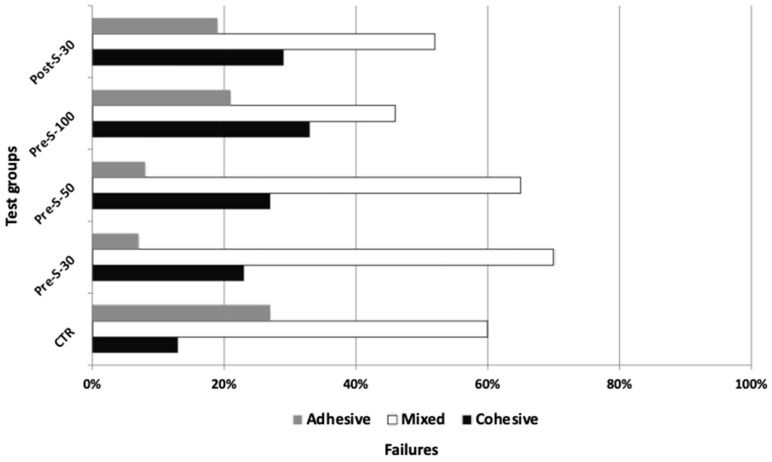
Failure analysis. A prevalence of mixed failures was observed in all experimental groups.

Regarding failure analysis, a prevalence of mixed failures was observed in all groups (Figure 5), ranging from 45% to 70%. The highest number of adhesive failures was observed for CTR, as well as the highest number of PTF.

**Figure 6:**
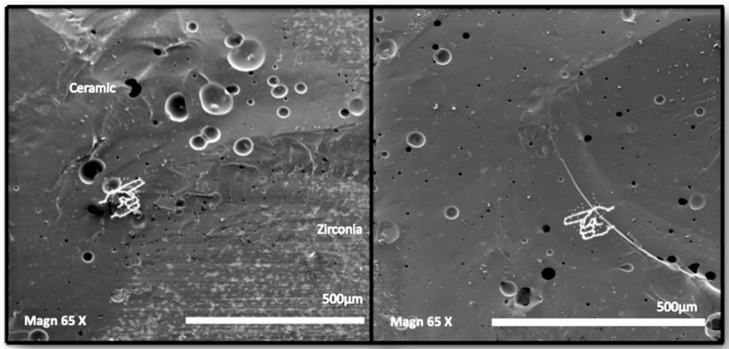
SEM photomicrographs showing a typical mixed failure at the veneering ceramic-zirconia interface (left side) and inner void defects within the heat-pressed ceramic layer (right side).

SEM photomicrographs of failures (65×) are presented in Figure 6. On the left side, the aspect of the most frequent and typical mixed failure with exposed zirconia covered by pressed ceramic is presented. The image on the right side reveals defects and voids within the heat-pressed ceramics itself.

No correlation was found between µTBS and Ra (*p* > 0.2).

## 4. Discussion

In this study, surface roughness and micro-tensile bond strength of veneering ceramic bonded to zirconia after different grit-blasting protocols were evaluated.

The hypotheses that different grit-blasting treatments with Al_2_O_3_ or silica-coated Al_2_O_3_ particles before or after sintering do not affect surface roughness or micro-tensile bond-strength of veneering ceramic bonded to zirconia have been rejected.

Regarding surface roughness, the present study revealed that grit-blasting the zirconia surface before sintering significantly increased surface roughness proportionally to the size of the airborne particles employed, this as compared to the control. Zirconia grit-blasted with 110-µm Al_2_O_3_ made the zirconia surface significantly rougher than all other grit-blasting protocols (and that of the control). No difference in Ra was recorded between the group grit-blasted with CoJet (3M Oral Care) after sintering and the control. The parameter Ra, which is the most common one reported in dental materials literature [29,30], was used in this study and represents the average roughness as measured by the profilometer. The lower Ra, the smoother the surfaces [31]. The results of this study corroborate those of other experimental studies that demonstrated that grit-blasting with Al_2_O_3_ particles enhances the surface roughness of zirconia [20,21,25]. However, most published data were obtained by carrying out grit-blasting with 50–110 μm alumina particles, and only a few of them used 30-μm silica-coated Al_2_O_3_ particles (CoJet, 3M Oral Care). Lassila et al. (2016) [32], demonstrated that airborne particle abrasion with Rocatec Soft (3M Oral Care) using 30-μm silica-coated Al_2_O_3_ particles, or with Rocatec Plus (3M Oral Care) using 105-μm silica-coated Al_2_O_3_ particles, or using 50-μm Al_2_O_3_ particles, significantly increased surface roughness. However, the same authors observed that the above-mentioned treatments may affect flexural strength, this depending on the flexural test and methodology used, as was also demonstrated by Nishigori et al. (2014) [20] and Guazzato et al. (2005) [21]. Harding et al. (2012) supported previous studies that revealed that sandblasting with alumina particles increased roughness [20,23] but may decrease flexural strength [23]. Valandro et al. (2021) stated that neither surface treatment of zirconia, nor thermocycling influences the porcelain-crack resistance or the resistance against delamination of bi-layered porcelain-veneered zirconia specimens [33].

Regarding micro-tensile bond strength, the highest µTBS was measured when pre-sintered zirconia was grit-blasted with CoJet (3M Oral Care) particles, thus combining the smallest 30-μm particle size with its peculiar silica-coating tribochemical effect. The better performance was significant when the statistical analysis was conducted including the pretesting failures (PTF), with the lowest recorded µTBS value or 0 MPa having been allocated to each PTF. However, a similar trend of increased bond strength measured upon pre-sintering grit-blasting was observed when the pretesting failures were excluded. Grit-blasting before sintering moderately increased bond strength, although the difference was not statistically significant. In literature, particularly in studies dealing with micro-tensile bond strength, the correct handling of specimens that failed before they could be tested, is still up for debate. By omitting the failures that failed prematurely during the µTBS test, only the non-failed specimens that exhibit the highest micro-tensile bond strength are counted in. This would lead to a bias toward a higher value (Figure 4). On the other hand, if failures are included as 0 MPa, judgment is too severe, since it is known that specimens were subjected to a certain but small tensile strength. Therefore, the statistical analysis performed including PTF’s with the lowest measured value allocated to each PTF was considered more appropriate.

The significant improvement in bond strength recorded when specimens were gritblasted with CoJet (3M Oral Care) after sintering was likely associated with the tribochemical effect of the silica-coated airborne particles, whereas the results obtained for the specimens grit-blasted before sintering were merely dependent on the roughness produced by grit-blasting.

The efficacy of CoJet (3M Oral Care) grit-blasting is related to the high kinetic energy of the SiO_2_-coated Al_2_O_3_ particles produced at impact and the fusion of silica with the substrate surface. The widely spread tribochemical silica-coating technique achieved using CoJet (3M Oral Care) [21,32] is claimed to provide micro-mechanical retention by embedding silica particles at the surface, hereby improving its chemical binding receptiveness [21,33,34]. 

Although it has extensively been demonstrated that silica-coating enhances bond strength of resin-based materials to zirconia [5,10,35,36,37,38,39], little is known about its likely positive effect on the adhesion of veneering ceramics to zirconia. No studies were found in the literature that compared the effect of airborne particle abrasion using Al_2_O_3_ particles versus silica-coated Al_2_O_3_ particles (CoJet or Rocatec Soft, both 3M Oral Care) on veneering ceramics-zirconia bonding. In fact, most of the studies evaluated only grit-blasting with 110-µm Al_2_O_3_ particles. Nishigori et al. (2014) reported that sandblasting with 50-µm Al_2_O_3_ did not significantly improve bond strength of veneering ceramic to zirconia. However, a decrease in bond strength was observed when the specimens were subjected to cycling loading [20]. Nakamura et al. (2009) and Liu et al. (2013) claimed that a blasting pressure in the range of 0.3–0.4 MPa with particle sizes of 50–70 µm improved bond strength without damaging the zirconia-surface structure [40,41]. Only He et al. (2014) measured the bond strength to zirconia treated with Al_2_O_3_ particles before sintering. They found that sandblasting before sintering at a pressure of 0.2 MPa significantly improved micro-mechanical interlocking and bond strength, as compared with specimens sandblasted after sintering [42]. In the same study, no difference was observed in bond strength of specimens treated using a higher blasting pressure of 0.4 MPa before or after sintering. Kim et al. (2011) demonstrated that the improved bond strength also depends on the greater contact area obtained by roughening, hereby also reducing interfacial failures [43]. However, controversial results have been reported by Fischer et al. (2008) and Harding et al. (2012), who demonstrated that sandblasting was not effective to improve adhesion at the veneering ceramic-zirconia interface or that it may even reduce the mechanical properties of zirconia [23,27]. Inokoshi et al. (2015) eventually demonstrated that sandblasting with CoJet (3M Oral Care) did not damage the zirconia surface and did improve bonding effectiveness and bond durability to zirconia [44,45].

Overall, micro-tensile and shear bond-strength approaches are used to test adhesion at interfaces. The results recorded in this study are in the same range of those reported in literature [19,20,26,27,46]. The mean bond strength at veneering ceramic-zirconia interfaces has been reported to be in a range varying from 22 to 45 MPa. Regrettably, to date there is no consensus regarding the type of test and the actual test conditions that are best used. Some authors performed shear bond-strength tests, while others employed micro-tensile bond-strength tests to evaluate bonding of veneering ceramic to zirconia. For this reason, research conducted using different methodologies and settings makes data comparison difficult. In this study, the micro-tensile bond-strength test was chosen because it has been demonstrated to be a more accurate tool to evaluate bonding effectiveness of veneering ceramics to zirconia [5,10,26,27,28,47]. However, the more easily conducted shear bond-strength test has been used most frequently. Nevertheless, a shear bond-strength test may lead to undesired stress-pattern distribution, inducing cohesive failures, and eventually erroneous data interpretation [10,27]. Despite the accuracy and effectiveness of the test methodology, when performing a micro-tensile bond-strength test, handling the brittle specimens is highly technique sensitive and involves very careful manipulation in order to avoid cutting defects or unexpected cracking of the micro-specimens (sticks). Using new sharp diamond saws at high cutting speeds and low loadings reduces vibrations and ensures finer cutting of the specimens.

On the basis of the results reported, it appears that a standard method to evaluate bond strength of veneering ceramic to zirconia with clinical relevance must still be developed.

SEM allowed to obtain a deeper insight into the surface topography produced by grit-blasting with respect to the μTBS data. The SEM photomicrograph in Figure 3a shows that grit-blasting drastically changed surface topography in the sense of enhanced potential for micromechanical interlocking and micro-retention. Detachment of zirconia particles and plastic deformation of the surface were also observed. EDS identified small fragments of alumina and silica on the grit-blasted surface. In particular, when the zirconia surface was grit-blasted with 50- or 110-µm Al_2_O_3_ particles before sintering, alumina appeared to have been embedded in the zirconia matrix and hence co-sintered (Figure 3b). When the zirconia surface was grit-blasted with 30-µm SiO_2_-coated Al_2_O_3_ after sintering, numerous fine silica particles were deposited on the zirconia surface (Figure 3c). These findings agree with those of Nagaoka et al. (2019), who characterized the ultrastructure and bonding properties of a tribochemical silica-coated zirconia [48].

**Figure 7:**
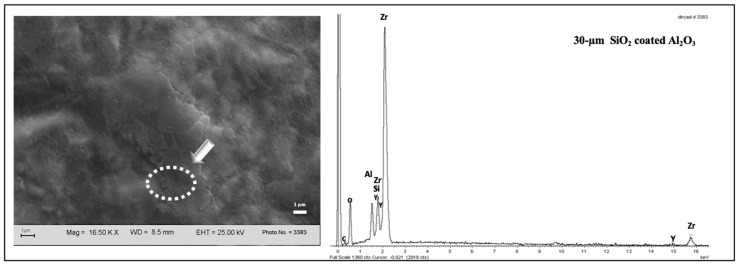
SEM photomicrograph of a pre-sintered specimen grit-blasted with 30-µm Cojet (3M Oral Care) powder (Post-S-30) and the corresponding EDS spectrum at the contact area. Peaks representing several elements of the veneering ceramic layer, among which Zr, Si, and Al, have been detected.

EDS also revealed chemical elements belonging to zirconia as well as veneering ceramics at the contact area (Figure 7). EDS point-analysis conducted on different points of the veneering ceramic-zirconia interface cross-sections revealed that grit-blasting with 30-µm SiO_2_-coated Al_2_O_3_ generated a reaction zone. These findings may suggest that grit-blasting with CoJet (3M Oral Care) not only produces widespread silica-particle depositioning on the surface but may also result in a partial zirconia-phase transformation, from tetragonal to monoclinic, and even lattice distortion. Although additional crystallographic studies are necessary, one may assume that this phenomenon induces a higher reactivity at the zirconia surface, thus modifying its interaction potential with veneering ceramic.

Nagaoka et al. (2019) characterized zirconia surfaces grit-blasted with Rocatec Soft powder (30-µm SiO_2_-coated Al_2_O_3_) and focused, in particular, on the effect on bond strength. They observed that the Al_2_O_3_ particles are irregular in shape and have sizes varying between 10 and 70 µm, and are coated with silica of around 50 nm or a thicker SiO_2_-particle layer [48]. Upon grit-blasting, alumina and silica was deposited on the zirconia surface but no real layer was coated. When silica-coated alumina particles hit the zirconia surface, their kinetic energy is partially converted into thermal energy, thus inducing a local increase of temperature and melting of silica particles that will adhere to the zirconia surface. However, several silica particles did not melt and were not strongly embedded into the zirconia surface but were merely spread over the surface. Moreover, some alumina particles fractured, causing fragments to remain attached onto the zirconia surface. Although the authors suggested that non-melted residual particles may interfere with bonding, a possible negative effect of surface-deposited silica and alumina particles on the bonding effectiveness to zirconia, among which they may also initiate cracks at the interface, has not been demonstrated yet. Despite further research is needed, these findings may explain why most of the failures observed in this study were mixed failures.

No correlation was found between surface roughness and bond strength, thus supporting the hypothesis that the high performance recorded for the pre-sintered specimens grit-blasted with CoJet (3M Oral Care) must more likely be attributed to the tribochemical effect than to the increased surface roughness/retention produced by grit-blasting. Furthermore, the application of the IPS e.max Zir-liner (Ivoclar) in a thin layer, as performed following the manufacturer’s instructions, may also have improved surface wettability and thus micromechanical interlocking efficiency between veneering ceramic and zirconia, as was reported before by Monaco et al. (year2014) [22] and Lassila et al. (year2013) [26].

Regarding failure analysis, overall, mostly mixed failures were observed, with pure interface failures having clearly been recorded less frequently. This finding could point to relatively good adhesion of veneering ceramic to zirconia. This mixed fracture pattern, as also observed in previous studies [20,26,27], was however often associated with a thin layer of ceramic that remained attached onto the zirconia framework. In this way, it may support the opinion of authors who assumed that the veneering ceramic remains the weakest point of by-layered all-ceramic restorations [27]. Feilzer et al. (2005 year) demonstrated that chipping failure started at the interface and that the interfacially initiated crack preferentially proceeds into the veneering ceramic layer due to the stiffness of zirconia. Moreover, the intrinsic brittleness of the veneering ceramic itself and the defects present within the veneering ceramic, as shown in Figure 6, may also trigger the veneering ceramic to chip. To conclude, determining the point of initial fracture is often very difficult, as already was claimed in previous investigations [5,10,27].

Additional zirconia-surface treatments, including acid etching, plasma treatment or application of a liner or glass coating, have been proposed to improve zirconia-bonding effectiveness, but the results are still inconclusive, by which further research is needed [22,23,25,26,49,50]. Despite a stable and predictable bonding of veneering ceramic to zirconia is essential for clinical success, the clinical occurrence of chipping and veneering ceramic delamination can seldom be correlated to outcomes of in-vitro studies [51]. Nevertheless, other factors, among which the coefficient of thermal expansion, design of the framework, occlusal loading, as well as patient and specific intra-oral factors, need also to be considered as well [5,10,12]. Probably most important to reduce crack occurrence/growth resulting in chipping of the veneering ceramic might be to select the proper veneering ceramic having a similar coefficient of thermal expansion as that of zirconia, as was done in this study.

## 5. Conclusions

Within the limitations of this study, it can be concluded that:

- A trend of increased surface roughness proportional to the size of the airborne particles employed was observed when grit-blasting was carried out before sintering.
- Grit-blasting with silica-coated alumina particles after sintering may improve the micro-tensile bond strength of veneering ceramic to zirconia.

Further investigations are needed, in particular to evaluate the effect of aging and the oral environment on the strength of the bond of veneering ceramics to zirconia, and on the long-term stability of this bond.

Besides, it is also important to determine a standardized method to evaluate bond strength at the veneering ceramic-zirconia interface, in particular to provide clinically relevant findings.

## Figures and Tables

**Table 1:** Materials tested in the study and their composition.

Materials	Composition	Coefficient of Thermal Expansion 10^−6^ K^−1^
IPS e.max ZirCAD, Ivoclar, Schaan, Liechtenstein	3Y-TZP, zirconium oxide (87–95 vol%), yttrium oxide (4–6 vol%), hafnium oxide (1–5 vol%), and alumina and silica (<1 vol%)	10.8 ± 0.3
IPS e.max Zir Liner, Ivoclar	Water, butandiol, and chloride	9.8 ± 0.3
IPS e.max ZirPress, Ivoclar	SiO_2_ with Li_2_O, Na_2_O, K_2_O, MgO, Al_2_O_3_, CaO, ZrO_2_, P_2_O_5_	9.8 ± 0.3

Data provided by the manufacturer.

**Table 2:** Grit-blasting treatments and application procedures.

Group—Surface Treatment	Working Distance	Working Time
**Pre-S-30**: 30-µm SiO_2_-coated Al_2_O_3_ before sintering	1 cm	15 s
**Pre-S-50**: 50-µm Al_2_O_3_ before sintering	1.5 cm	15 s
**Pre-S-110**: 110-µm Al_2_O_3_ before sintering	1.5 cm	15 s
**Post-S-30**: 30-µm SiO_2_-coated Al_2_O_3_ after sintering	1 cm	15 s
CTR—no treatment	-	-

**Table 3:** Micro-tensile bond strength (µTBS in MPa) and failure mode of specimens.

Group—Surface Treatment	PTF/N	µTBS (MPa) Mean (SD)	Failure Patterns		
			Cohesive	Mixed	Adhesive
CTR-No treatment	12/48	20.2 (11.2) ^B^	13%	60%	27%
Pre-S-30: 30-µm SiO_2_-coated Al_2_O_3_-pre-sintering	6/48	21.8 (10.4) ^B^	23%	70%	7%
Pre-S-50: 50-µm Al_2_O_3_-pre-sintering	5/48	24.1 (12.6) ^B^	27%	65%	8%
Pre-S-110: 110-µm Al_2_O_3_-pre-sintering	8/48	26.4 (14.1) ^B^	33%	46%	21%
Post-S-30: 30-µm SiO_2_-coated Al_2_O_3_-post-sintering	3/48	28.5 (12.6) ^A^	29%	52%	19%

Different superscript letters indicate statistically significant differences (*p* < 0.05). Data reported in this table refer to the analysis conducted by including PTF (pretesting failures) with the lowest obtained value (‘PTF = MIN VALUE’). ‘N’ indicates the total number of specimens for each group tested.

## Data Availability

The raw data supporting the conclusions of this article will be made available by the authors on request.
